# Estimating the annual entomological inoculation rate for *Plasmodium falciparum* transmitted by *Anopheles gambiae s.l.* using three sampling methods in three sites in Uganda

**DOI:** 10.1186/1475-2875-13-111

**Published:** 2014-03-21

**Authors:** Maxwell Kilama, David L Smith, Robert Hutchinson, Ruth Kigozi, Adoke Yeka, Geoff Lavoy, Moses R Kamya, Sarah G Staedke, Martin J Donnelly, Chris Drakeley, Bryan Greenhouse, Grant Dorsey, Steve W Lindsay

**Affiliations:** 1 Infectious Diseases Research Collaboration, Kampala, Uganda; 2 Department of Epidemiology, Johns Hopkins University, Baltimore, USA; 3 London School of Hygiene and Tropical Medicine, London, UK; 4 Department of Medicine, Makerere University College of Health Sciences, Kampala, Uganda; 5 Department of Vector Biology, Liverpool School of Tropical Medicine, Pembroke Place, Liverpool, UK; 6 Department of Medicine, University of California, San Francisco, USA; 7 School of Biological and Biomedical Sciences, Durham University, Durham, UK

**Keywords:** Malaria, *Plasmodium falciparum*, *Anopheles gambiae s.l*, Uganda, Entomological inoculation rate, Human-landing catches, CDC light traps

## Abstract

**Background:**

The *Plasmodium falciparum* entomological inoculation rate (*Pf*EIR) is a measure of exposure to infectious mosquitoes. It is usually interpreted as the number of *P. falciparum* infective bites received by an individual during a season or annually (a*Pf*EIR). In an area of perennial transmission, the accuracy, precision and seasonal distribution (i.e.*,* month by month) of a*Pf*EIR were investigated. Data were drawn from three sites in Uganda with differing levels of transmission where falciparum malaria is transmitted mainly by *Anopheles gambiae s.l.* Estimates of a*Pf*EIR derived from human-landing catches – the classic method for estimating biting rates – were compared with data from CDC light traps, and with catches of knock down and exit traps separately and combined.

**Methods:**

Entomological surveillance was carried out over one year in 2011/12 in three settings: Jinja, a peri-urban area with low transmission; Kanungu, a rural area with moderate transmission; and Nagongera, Tororo District, a rural area with exceptionally high malaria transmission. Three sampling approaches were used from randomly selected houses with collections occurring once a month: human-landing collections (eight houses), CDC light traps (100 houses) and paired knock-down and exit traps each month (ten houses) for each setting. Up to 50 mosquitoes per month from each household were tested for sporozoites with *P. falciparum* by ELISA. Human biting rate (HBR) data were estimated month by month. *P. falciparum* Sporozoite rate (*Pf*SR) for yearly and monthly data and confidence intervals were estimated using the binomial exact test. Monthly and yearly estimates of the HBR, the *Pf*SR, and the *Pf*EIR were estimated and compared.

**Results:**

The estimated a*Pf*EIR values using human-landing catch data were 3.8 (95% Confidence Intervals, CI 0-11.4) for Jinja, 26.6 (95% CI 7.6-49.4) for Kanungu, and 125 (95% CI 72.2-183.0) for Tororo. In general, the monthly *Pf*EIR values showed strong seasonal signals with two peaks from May-June and October-December, although the precise timing of the peaks differed between sites. Estimated HBRs using human-landing catches were strongly correlated with those made using CDC light traps (r^2^ = 0.67, p < 0.001), and with either knock-down catches (r^2^ = 0.56, p < 0.001) and exit traps (r^2^ = 0.82, p < 0.001) or the combined catches (r^2^ = 0.73, p < 0.001). Using CDC light trap catch data, the *Pf*SR in Tororo was strongly negatively correlated with monthly HBR (r^2^ = 0.44, p = 0.01). In other sites, no patterns in the *Pf*SR were discernible because either the number *P. falciparum* of sporozoite positive mosquitoes or the total number of mosquitoes caught was too low.

**Conclusions:**

In these settings, light traps provide an alternative method for sampling indoor-resting mosquitoes to human-landing catches and have the advantage that they protect individuals from being bitten during collection, are easy to use and are not subject to collector bias. Knock-down catches and exit traps could also be used to replace human-landing catches. Although these are cheaper, they are subject to collector bias.

## Background

The intensity of malaria transmission by mosquitoes is central to efforts to control and eradicate malaria, and various methods to estimate it have been developed over the past 80 years. The pre-eminent method for estimating transmission entomologically has been human-landing catches, where mosquitoes are caught as they attempt to land on the exposed limbs of field workers [1,2]. Human-landing catches are regarded as the ‘gold standard’, largely based on *a priori* arguments about the validity of the method in that it represents natural transmission dynamics, and the method has been used in many studies to estimate the entomological inoculation rate (*Pf*EIR), the number of infective bites received by an individual over a defined time period [3].

CDC light traps provide one alternative method for estimating biting rates and comparisons between human-landing catches and light traps have been made in several studies (Table 1). Typically traps are positioned indoors next to a person sleeping under a treated bed net. The trap collects mosquitoes frustrated in their efforts to feed on people in the room and reduces the number of bites a person would receive from vectors. Moreover all individuals in the room are protected from mosquitoes because they are sleeping under nets. Their disadvantage is that the traps collect few mosquitoes outdoors [4], are relatively expensive, and require a charged 6 V battery to function.

**Table 1:** Studies using light trap collections and human-landing catches

**Site**	**Major vector**	**Bait**	**Date of study**	**Relative catching efficiency of light trap collections**	**Reference**
Bobodioulasso, Burkina Faso	*An. gambiae s.l.*	Unprotected sleepers	1968/9	46%^a^	[5]
Brazzaville, Republic of the Congo	*An. gambiae s.s.*	No sleeper	1971	98%^a^	[6]
Nr Kisumu, Kenya	*An. gambiae s.l.*	Unprotected sleeper	1971/2	No comparison made	[7]
Bignona, Senegal	*An. gambiae s.l.*	Unprotected sleeper	1984/6	91%^b^	[8]
Nr Muheza, Tanzania	*An. gambiae s.l.*	Sleeper under an untreated net	1986/8	150%^b^	[9]
Mbébé, South Cameroon	*An. gambiae s.l.*	Unprotected sleeper	1989/90	25%^b^	[10]
Nr Bagamoyo, Tanzania	*An. gambiae s.l.*	Sleeper under an untreated net	1992	123%^b^	[11]
Nr Ougadougou, Burkina Faso	Mainly *An. arabiensis*	Sleeper under an untreated net	1992/3	108%^b^	[12]
Dar es Salaam, Tanzania	*An. gambiae s.l.*	Sleeper under a treated or untreated net	2008	5%^b^	[13]
Macha, Zambia	Mainly *An. arabiensis*	Sleeper under a treated or untreated net	2007/9	96%^b^	[14]
Ahero rice irrigation scheme, Kenya	*An. arabiensis*	Sleeper under an untreated net	2002	60%	[15]

^a^house-resting collections the following morning.

^b^human-landing catches indoors.

Another common method used for sampling indoor mosquitoes is using knock-down catches and exit traps used alone or combined [1,2]. Spraying insecticides indoors early in the morning is an activity normally appreciated by householders since it reduces the number of mosquitoes (and other insects) in the house. Exit traps are placed in windows and the combined collections of blood-fed mosquitoes made indoors and in the exit trap used to estimate potential biting rates. The disadvantage of this method is that the exit traps are bulky and are difficult to transport in large numbers, and importantly, are subject to collector bias.

In this study, malaria transmission intensity was estimated by the annual *Plasmodium falciparum* entomological inoculation rate (a*Pf*EIR), using these three different methodologies: human-landing catches (the gold standard), CDC light traps, and pyrethrum spray catches alone or combined with exit trap collections. This was done specifically to determine whether human-landing catches could be replaced with one of the alternative collection techniques for routine entomological surveillance. The data made it possible to investigate the accuracy and precision of a*Pf*EIR and its seasonal distribution (i e*,* month by month). The study was conducted in three different study sites, representing markedly different ecologies: Jinja town in south-eastern Uganda, Kanungu village in western Uganda, and Nagongera village in Tororo District, eastern Uganda [16].

The information collected in this study will complement and support other studies that describe the clinical pattern of infection and morbidity, as well as the level of anti-malarial drug resistance in the parasite populations and insecticide resistance in local vector populations, at the same sites. The collection and integration of these diverse data sets will characterize malaria in the study sites and establish a robust framework for developing future interventions against this disease.

## Methods

### Study site

**Figure 1:**
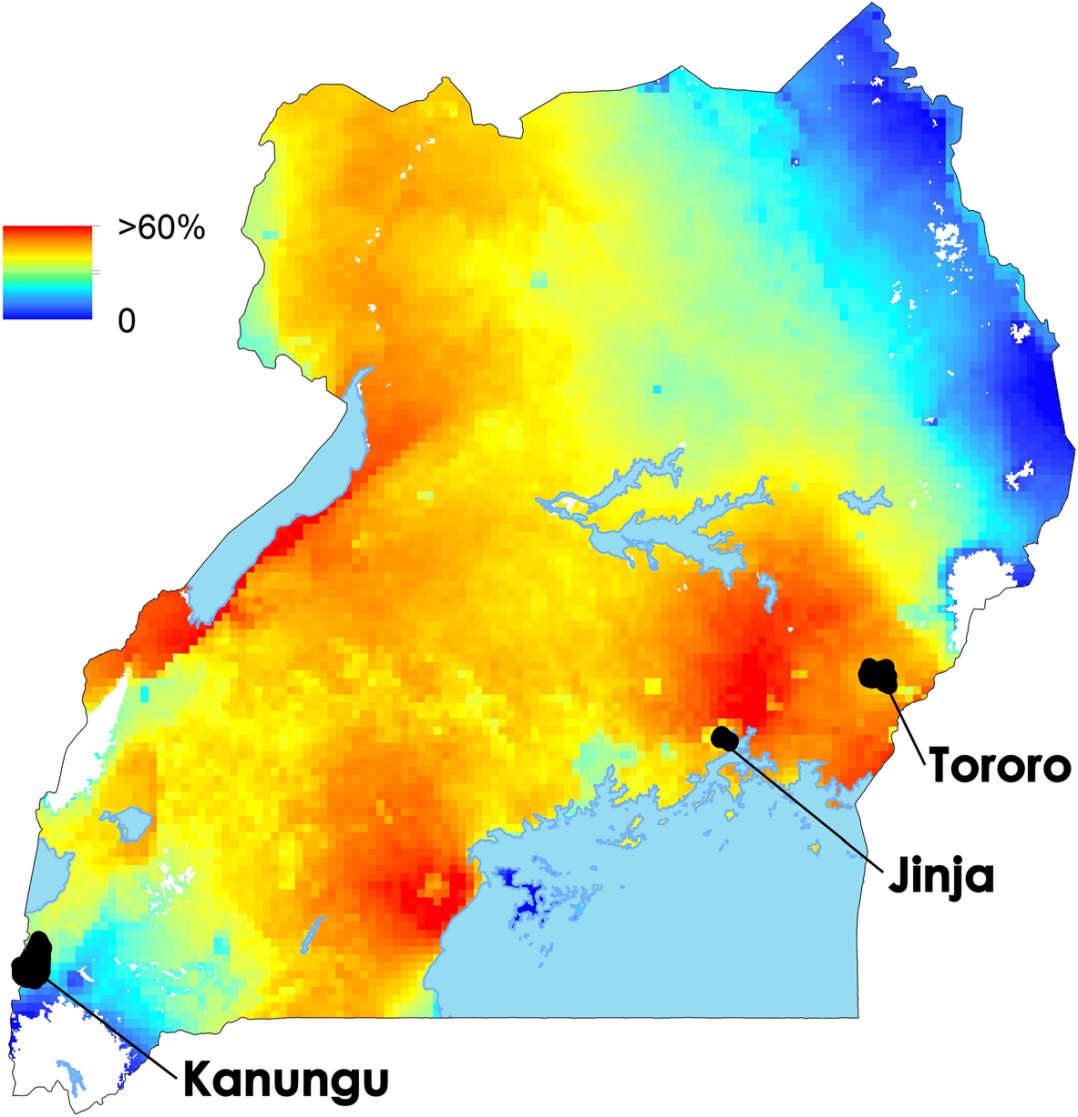
**Map of Uganda showing study sites.** The colours are *Pf*PR in the over two and up to ten-year age group from the MAP 2010 dataset [15].

Studies were carried out in Walukuba subcounty, Jinja District (00° 26′ 33.2″ N, 33°13′ 32.3″ E); Kihihi subcounty, Kanungu District (00°45′ 03.1″ S, 29°42′ 03.6″ E); and Nagongera subcounty, Tororo District (00°46′ 10.6″, N 34°01′ 34.1″ E) (Figure 1). Jinja is in the southeast region at an elevation of 1,215 m above sea level and the study site is peri-urban, close to a swampy area near Lake Victoria. The major malaria vector species here was *Anopheles gambiae s.s.* ten years ago [16], but it is now *Anopheles arabiensis*[17]. Kanungu is a rural area of rolling hills in western Uganda located at an elevation of 1,310 m above sea level, where farmers grow bananas, millet, rice, cassava, potatoes, sweet potatoes, tomatoes, maize, groundnuts, and beans. The main vector here is *An. gambiae s.s.*. Tororo is located in the eastern region at an elevation of 1,185 m above sea level in an area of savannah grassland interrupted by bare rocky outcrops and low-lying wetlands, where maize, rice, cassava, sweet potatoes, sorghum, groundnuts, soya beans, beans, and millet are cultivated. The major malaria vector species reported for the region are *Anopheles gambiae s.s.* and *Anopheles funestus* with small numbers of *An. arabiensis*[16,18]. There are typically two rainy seasons in Uganda (March to May and August to October) with annual rainfall of 1,000-1,500 mm.

### Entomological surveillance

Prior to conducting the entomological surveys, all households within each subcounty were enumerated and mapped to generate a sampling frame for the random selection of houses representative of the catchment areas (Walukuba, Jinja 9,881 households; Kihihi, Kanungu 12,774 households; Nagongera, Tororo 6,992 households).

### Human-landing catches

Human-landing catches were conducted indoors and outdoors in and around eight randomly selected houses at each site each month. At each site, two different houses were selected each night, at least 300 m apart, for four consecutive nights. Thus, all eight households were sampled in the same week each month. Catches were designed to replicate normal human subject behaviour, assuming many residents will be outdoors in the early evening, and that most will retire to bed before 22.00. At each house two adults were stationed outdoors 10 m from the house, and two were stationed indoors. Outdoor collections were conducted from 18.00 to 21.50, after which time few people are outdoors, and indoor collections from 18.00 to 05.50. Field workers collected mosquitoes landing on their exposed legs using aspirators and torchlight for 50 min, with a 10-min break each hour. They were rotated between sites on different nights.

### Light trap collections

Light traps were positioned indoors next to a child aged six months to ten years, sleeping under a long-lasting insecticidal net (LLIN) in 100 households randomly selected at each site and collections made monthly using miniature CDC light traps (Model 512; John W. Hock Company, Gainesville, FL, USA). The traps were positioned with the light bulb 1 m above the floor at the foot end of the bed where a person slept under a LLIN. Traps were set at 19.00 and collected at 07.00 the following morning. If it was not possible to set the trap in the intended house, it was moved to the nearest similar house. If the occupant did not spend the night in the selected room or if the trap was faulty, the data were excluded from the analysis. Each night approximately 12 traps were set for four nights each week. They were rotated in the same order each month.

### Pyrethrum spray and exit trap collections

Bedrooms in which an exit trap was positioned over a window during the previous evening were sprayed the following morning using a non-residual pyrethroid (BOP, McBride Caribbean Ltd). Muirhead-Thomson type exit traps [19] made from cotton mosquito netting placed over a metal wire frame (40 × 40 × 40 cm) were placed over the windows of the houses to capture any escaping mosquitoes. In each site, ten households were randomly selected for the spray collections and sampled monthly. Pyrethrum spray collections took place between 07.00 and 09.00. Food and water was removed from the house and white sheets spread on the floor and over the furniture in the house. Two field workers, one inside the house and one outside, sprayed around the eaves with a non-residual pyrethroid. The field worker inside the house then sprayed the roof and walls. The house was closed for 10 min after which the white sheets were brought outside (where there is sufficient light to see the dead and dying mosquitoes), and dead mosquitoes collected from the sheets and transferred to the field laboratory on moist filter papers in Petri dishes for identification and processing. To collect house-leaving mosquitoes, window exit traps were set at 18.00 and collected between 06.00 and 07.00 the following morning.

### Processing of mosquito specimens

All anophelines were identified taxonomically to species level where possible. Identification of anophelines was based on morphological criteria according to established taxonomic keys [20,21]. Identification of members of the *An. gambiae* complex was assessed by PCR for 30 mosquitoes randomly selected at each site, each month [22]. *P. falciparum* sporozoites were identified in individual mosquitoes stored on desiccant using an ELISA technique [23].

### Data management

Entomological data were recorded by field workers on standardized data forms. The forms were double-entered by two data entry clerks. The first and second entry datasets were combined and errors corrected to produce a single dataset. This was submitted to consistency checking by generic and study-specific algorithms designed to identify sources of error. When inconsistencies were found, they were checked against the original forms and subsequently amended in the dataset.

### Analytical plan

#### Human biting rates

Human biting rates (HBR) measured directly from human-landing collections made indoors were compared with the number of mosquitoes collected using CDC light traps and knock-down catches and exit traps. Confidence intervals on the HBR were computed using two methods. First, the samples were bootstrapped and the 2.5^th^ and 97.5^th^ quantiles used for the confidence limits. Second, conventional methods (i.e*,* the mean ± 1.96 standard errors) based on the central limit theorem were used to compute confidence intervals.

#### Sporozoite rates

The *P. falciparum* sporozoite rate (*Pf*SR) is the number of mosquitoes infected with sporozoites divided by the total number of mosquitoes examined using each respective method of mosquito collection, expressed as a percentage. A stopping rule was deployed, for the practical reason of limiting expenses, so that a maximum of 50 mosquitoes were tested from any trap in any site which caught more than this amount. Confidence limits on the *Pf*SR were computed for each month, for each household, and for the whole year. *Pf*SR data were compared with HBR data for associations with the month, the household and the number of mosquitoes caught.

#### Entomological inoculation rates

*Pf*EIR is conventionally computed by taking the product of the daily HBR, the *Pf*SR from the caught mosquitoes, and 365, the number of days in the year. To introduce the computation of the *Pf*EIR for this study, let *M*_*h*,*m*_ denote the number of mosquitoes that were caught from each house (*h*) in each month (*m*), *N*_*h*,*m*_ the number of mosquitoes that were examined for *P. falciparum* infection, and *Z*_*h*,*m*_ the number of these that were *P. falciparum* sporozoite positive. If every mosquito had been tested for *P. falciparum* sporozoites (i e*,* if *M*_*h*,*m*_ = *N*_*h*,*m*_ for all *h* and *m*), then the *Pf*EIR would be given by

$$\frac{365}{\left|h\times m\right|}\sum_{h,m}Z_{h,m}$$

If some mosquitoes are subsampled for the presence of *P. falciparum* sporozoites (i.e *.,* if*M*_*h*,*m*_ < *N*_*h*,*m*_ for some samples), then a general formula for the *Pf*EIR is the following:

$$\frac{365}{\left|h\times m\right|}\left[\sum_{h,m}Z_{h,m}+\sum_{N_{h,m}<M_{h,m}}p_{h,m}\left(M_{h,m}-N_{h,m}\right)\right]$$

where *p*_*h*,*m*_ is the *Pf*SR applied to the untested mosquitoes for that household and month, and where |*h* × *m*| is the number of daily samples. Here, the overall *Pf*SR for all samples was used,

$$p_{h,m}=\sum_{h,m}\frac{Z_{h,m}}{N_{h,m}}.$$

A subsequent paper will compare the results of using alternative statistical methods for calculating *Pf*EIR.

Since the *Pf*EIR estimates were produced by different catching methods, and since the subsequent computation involved a mixture of quantities described by different probability distribution functions, confidence intervals describing the precision of the estimates were generated two different ways, using bootstrapping and conventional methods as described for the estimation of confidence intervals for the human biting rate.

### Ethical issues

Written informed consent from the head of household or an adult household representative was obtained by the field worker prior to conducting surveillance in a household. Field workers provided written informed consent for the human-landing catches, were paid for their work and provided with malaria chemoprophylaxis, consisting of mefloquine (250 mg tab orally once weekly) or doxycycline (100 mg tab orally each day). They were also offered medical treatment for any illness that developed during the period of their employment.

Ethical approval for this study was provided by the Uganda National Council for Science and Technology, the Makerere University School of Medicine Research and Ethics Committee, the University of California, San Francisco Committee on Human Research, London School of Hygiene and Tropical Medicine ethical committee and the School of Biological and Biomedical Sciences Ethics Committee, Durham University.

## Results

### Species composition

Monthly collections were performed at all three sites over a 12-month period from October 2011 to September 2012. A total of 2,286 female *Anopheles* were collected using human-landing catches, 66,476 using light traps and 2,470 using knock-down catches and exit traps. Of these, 88.5% were *An. gambiae s.l*. in Jinja, 99.8% in Kanungu, and 93.5% in Tororo based on light trap collections. Of the members of the *An. gambiae* complex tested, 36.3% were *An. gambiae s.s.* and 63.7% *An. arabiensis* in Jinja, 99.2% *An. gambiae s.s.* and 0.8% *An. arabiensis* in Kanungu, and 81.5% *An. gambiae s.s.* and 18.5% *An. arabiensis* in Tororo. In all three sites *An. gambiae s.l.* were far more common than *An. funestus*, with the greatest numbers of *An. gambiae s.l.* collected in Tororo, followed by Kanungu and Jinja.

### Human biting rates

**Figure 2:**
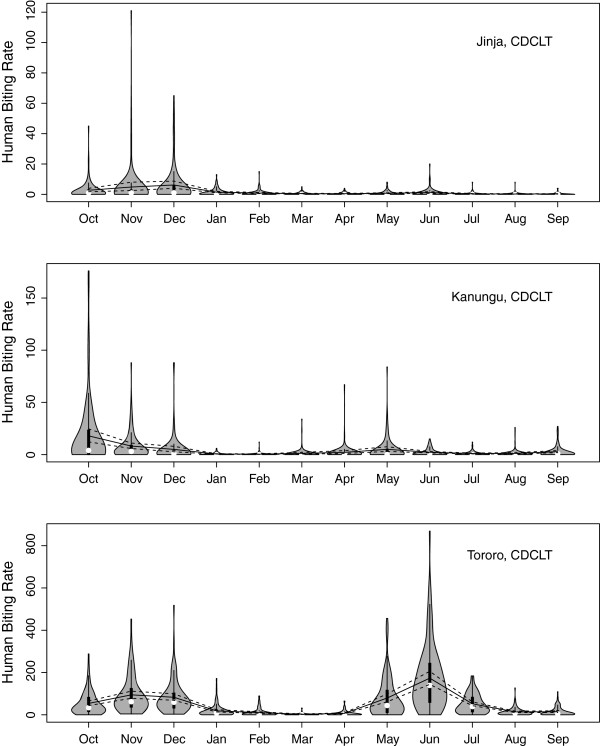
Violin plot of daily human biting from CDC light traps, plotted month by month, with the mean and confidence intervals (solid and dashed lines respectively).

The estimated HBR were different in each site and by each method (Table 2), with Tororo being the highest, Kanungu being intermediate and Jinja being the lowest. The estimates also differed strongly by method, with light traps catching the most, human-landing catches being intermediate, and the catches from exit traps and pyrethroid spray catches being the lowest per catching effort. Confidence intervals using bootstrapped values and standard formulae gave nearly identical results. The HBR had a strong, seasonal signal (Figure 2) that differed slightly across the three sites. Jinja had a major peak in November and a minor peak in June. Kanungu had a major peak in October and a minor peak in May; and Tororo had its major peak in June with a minor peak in December.

**Table 2:** The annual human biting rates, sporozoite rates, and the annual entomological inoculation rates reported for all three sites and by all three methods for catching mosquitoes, and for the combined data

**Parameter**	**Sampling method**	**Sampling site**		
		**Jinja**	**Kanungu**	**Tororo**
aHBR (95% CI)	HLC	270 (171-391)	1,022 (433-1,859)	7,399 (5,349-9,726)
	LT	605 (485-744)	1,460 (1,208-1,736)	18,359 (16,711-20,133)
	KDC and ET	94 (52-149)	849 (487-1,278)	6,570 (5,010-8,255)
	All	537 (434-656)	1,377 (1,155-1,621)	16,606 (15,133-18,094)
*Pf*SR (95% CI) (numerator/denominator)	HLC	1.4% (0.03-7.5%) (1/71)	2.6% (1.1-5.3%) (7/269)	1.7% (1.2-2.4%) (33/1,946)
	LT	0.3% (0.6-1%) (11/1,812)	1.2% (0.9-1.7%) (39/3,069)	1.9% (1.7-2.0%) (531/28,076)
	KDC and ET	0 (0-11%) (0/31)	0.35% (0-1.2%) (1/279)	2.5% (1.9-3.3%) (54/2160)
	All	0.6% (0.3-1%) (12/1,914)	1.3% (1.0-1.7%) (47/3,617)	1.9% (1.8-2.1%) (618/32,182)
a*Pf*EIR (95% CI)	HLC	3.8 (0-11.4)	26.6 (7.6-49.4)	125 (72.2-183)
	LT	3.49 (1.59-5.70)	14.4 (9.3 -20.0)	340 (290-394)
	KDC and ET	0	3.04 (0-9.1)	164 (113-216)
	All	3.2 (1.6-5.1)	14.2 (9.8-19.2)	310 (267-356)
Total number of sampling days	HLC	96	96	96
	LT	1,151	1,163	1,191
	KDC and ET	120	120	120
	All	1,367	1,379	1,407

aHBR: annual human biting rate; *Pf*SR: *Plasmodium falciparum* sporozoite rate; a*Pf*EIR: annual *Plasmodium falciparum* entomological inoculation rate.

### *P. falciparum* Sporozoite rates

**Figure 3:**
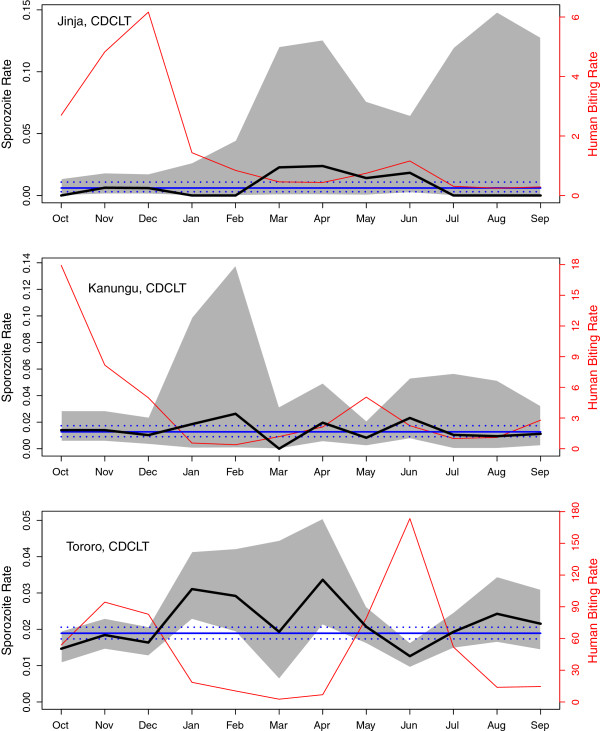
***P. falciparum *Sporozoite rates by month for the mosquitoes in the CDC light traps (thick black) with the confidence intervals (shaded in grey) by the exact test.** The mean annual *Pf*SR and confidence intervals are also plotted (blue solid, and dashed). The mean monthly HBR is plotted (red). In Tororo, there is a statistically significant negative correlation between the mean HBR and the *Pf*SR.

The *Pf*SR differed among sites for the light trap data (Wilcoxon matched pairs, signed ranks test, p <0.001) and for the combined data (p < 0.01), but not for the human-landing catches (p = 0.55) and was of borderline significance for the spray collections and exit traps combined (p = 0.051; Table 2). Similarly, the *Pf*SR did not differ by method for any of the sites, although the p-values bordered on being significant for Kanungu (Jinja, p = 0.64; Kanungu, p = 0.06; Tororo, p = 0.11). *Pf*SR for the light traps varied seasonally in Tororo (p < 0.001), and they were negatively correlated with the HBR (r^2^ = 0.44, p = 0.01 on the slope), but there was no apparent seasonal signal for Jinja or Kanungu, perhaps because the total number of *P. falciparum* sporozoite positive mosquitoes caught was so low (Figure 3). Associations were also sought between the *Pf*SR by household and for those houses where more than 50 mosquitoes were caught. In Tororo, the *Pf*SR patterns appeared to be negatively correlated with the HBR values.

### Entomological inoculation rates

**Figure 4:**
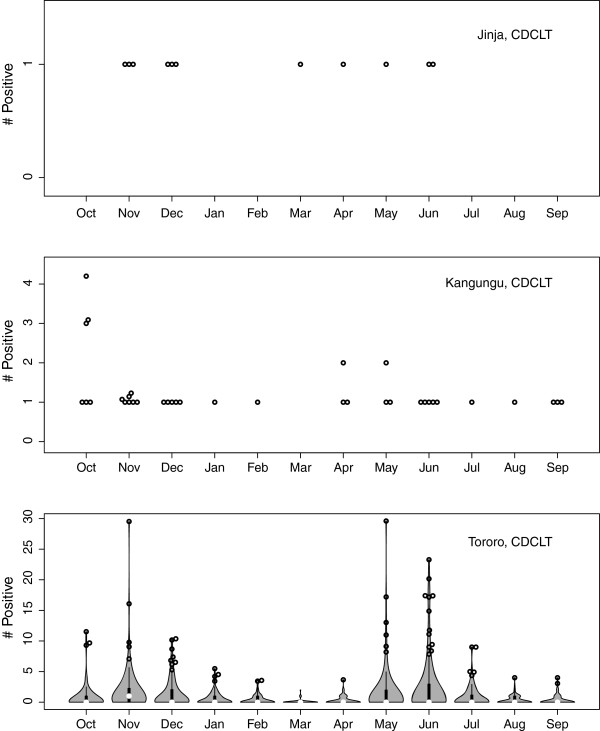
**The number of *P. falciparum* sporozoite positive mosquitoes from daily trapping with CDC light traps, plotted month by month.** For Jinja and Kanungu, the data are presented as a beeswarm plot of only the positive mosquitoes caught in each month (i e, *P. falciparum* sporozoite negative mosquitoes are not shown). For Tororo, the data are shown as a violin plot of the number of positive mosquitoes (i e, *P. falciparum* sporozoite positive and negative are plotted). The extreme values have been shown as a beeswarm.

Annual *Pf*EIR clearly differed by site for all methods, and the *Pf*EIR generated by the various methods all differed from one another within a site (Table 2). Too few infectious mosquitoes were caught in Jinja and Kanungu to be confident about any seasonal patterns in *Pf*EIR. The *Pf*EIR in Tororo, however, was clearly seasonal with a pattern that was similar to the seasonal HBR pattern, with two peaks, one around November and the other around June (Figure 4).

### Comparison of catching methods

**Figure 5:**
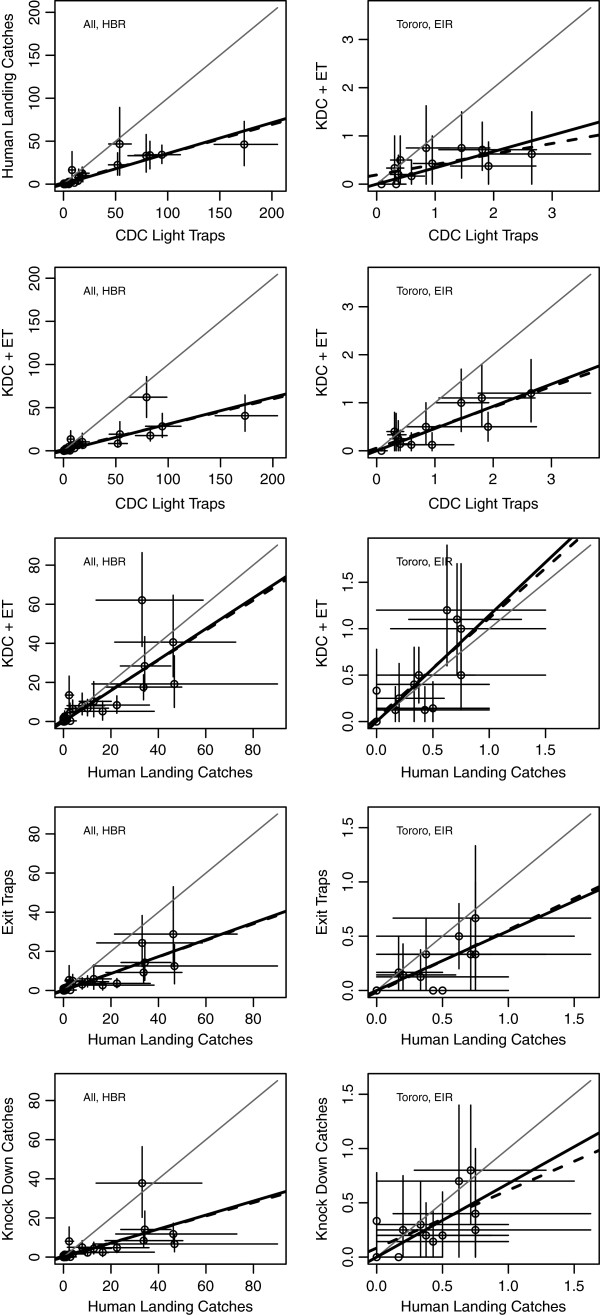
**Pair-wise comparisons by method of the number of mosquitoes caught each month from all sites (top row) and the monthly *Pf*EIR from Tororo (bottom row).** The grey line represents a perfect one-to-one correlation, the thick black line represents the line of best fit for the rooted regression and the thick dashed line that for the unrooted regression. The slopes (*m*), intercepts (*b*), p-values, and r^2^ values from linear regression are shown in Table 3.

Human-landing catches were correlated with light trap collections and those made using knock-down collections and exit traps separately and combined, across all three sites or for Tororo alone (Figure 5). Linear regression was done both rooted and unrooted. In all cases, the slope was statistically significant, but the intercept was not. The rooted relationship, therefore, seems to be a better model as expected since logically one would expect when no mosquitoes were collected by human-landing catches there would be none caught in a light trap. The slopes of the relationships varied, however, by method (Table 3, Figure 5). The knock-down collections and window traps are natural complements: all mosquitoes were present and could have blood fed, but any particular mosquito would have either exited the house or knocked down and collected the next morning. Both methods were highly correlated with HLC, but the combined catches were most similar (*i.e.* a slope of .79 vs. .43 for ET or .36 for KDC).

**Table 3:** **The results of the linear regression analysis shown in Figure **5

**Parameter**	**x**	**y**	**Rooted**			**Unrooted**				
			***m***	**p-val**	**r**^**2**^	***m***	**p-val**	***b***	**p-val**	**r**^**2**^
All (HBR)	LT	HLC	0.36	<0.001	0.85	0.34	<0.001	1.60	0.160	0.81
	LT	KDC&ET	0.31	<0.001	0.75	0.29	<0.001	1.40	0.320	0.69
	HLC	KDC&ET	0.79	<0.001	0.73	0.76	<0.001	0.79	0.597	0.66
	HLC	ET	0.43	<0.001	0.82	0.42	<0.001	0.32	0.616	0.77
	HLC	KDC	0.36	<0.001	0.56	0.34	<0.001	0.48	0.634	0.47
Tororo (*Pf*EIR)	LT	HLC	0.34	<0.001	0.76	0.22	0.025	0.19	0.093	0.41
	LT	KDC&ET	0.46	<0.001	0.87	0.43	0.001	0.06	0.618	0.70
	HLC	KDC&ET	1.10	<0.001	0.80	1.10	0.009	0.04	0.811	0.52
	HLC	ET	0.55	<0.001	0.77	0.57	0.009	-0.02	0.862	0.52
	HLC	KDC	0.68	<0.001	0.74	0.53	0.039	0.08	0.461	0.36

The results give the slopes (*m*), intecepts (*b*), p-values and r^2^ values for the rooted and unrooted linear analysis. The r^2^ values suggest very good correlations among all metrics, though the slopes are generally not close to one, suggesting all methods are biased relative to one another.

## Discussion

A long-standing goal for mosquito-borne pathogens has been the development of reliable entomological metrics of exposure and transmission [3,24]. These efforts have evolved over more than 80 years into a set of methods for estimating the a*Pf*EIR [2,24-26].

A persistent issue has been the accuracy and precision of methods for estimating the *Pf*EIR, the HBR, or local mosquito population density and mosquito bionomic parameters. All these metrics are influenced by the methods used for catching mosquitoes, the details of the study protocols, and the local properties of the vectors and their behaviour and ecology. Among the prominent methods are estimates of the HBR using human-landing catches, CDC light traps, or knock-down catches combined with exit traps; or alternatively, estimates of the mosquito population density using mark-release-recapture studies. Despite hundreds of studies conducted so far, questions remain about the accuracy of these methods, which can only be addressed by extensive cross-validation.

The present study shows that there was a strong correlation between collections made with human-landing catches and either light traps or the combination of knock-down collections and exit traps. Under the assumption that zero catches with human-landing catches corresponded to zero catches with light traps, over 73% of the variation in mosquito numbers was explained by the direct relationship between human-landing catches and either of the other two sampling methods. It should though be appreciated that human-landing catches are unlikely to perfectly match the actual biting rate experienced by people living in a community since nobody sits up all night with the lower limbs exposed, and catching efficiency is dependent both on the skill and alertness of the collector. Recognizing the limitations of the human-landing catches for estimating the HBR could indicate that both light traps and knock-down catches/exit traps combined may actually be better at estimating the true HBR than suggested by the values of r^2^ reported.

Several studies have assessed the relative catching efficiency of light traps for sampling *An. gambiae* s.l. (Table 1). In most cases, as in the present study, there is a reasonable correspondence between light trap collections and either resting collections or human-landing catches. Indeed, the CDC light trap catches in this study generally caught more mosquitoes than other methods. However, light traps were clearly not effective in Dar es Salaam, and the authors comment that this may be because the study area was well lit at night [13]. In most studies light traps were used where people slept under untreated bed nets, but in this case study participants slept under permethrin-treated nets. Although there have been concerns raised that treated nets may repel mosquitoes, studies have shown that in practice using LLINs has little or no impact on collections made from light traps [27].

No other studies could be found that compared human-landing catches with knock-down catches and exit traps together. These results indicate that knock-down catches combined with exit traps were consistent with human-landing catches, although the results from Jinja indicate that they may not be so effective where vectors occur in low densities. Thus this method represents an alternative to light traps. Nonetheless, whilst this technique may be cheaper to operate than light traps, exit traps are bulky and therefore difficult to transport.

Visual inspection of the catch data describing the HBR and the *Pf*EIR were approximately negatively binomially distributed in most months. This is typical for insect distributions [28,29], including biting rates for *An. gambiae s.l.*[30,31]. It implies that a relatively small proportion of people are at a high risk of infection, whilst for most people the risk is relatively low or moderate. Thus at low biting densities there can be marked variation in malaria infection across a small area [32,33]. Peak biting rates occurred in May/June and October to December at the different study sites. This corresponds with the periods at the end of the two rainy seasons, when vector populations have expanded progressively during the rainy season.

The *Pf*SR was calculated and several different methods were used to determine the 95% confidence intervals, but there was little difference between methods. Interestingly, in Tororo monthly *Pf*SR were negatively correlated with HBR. This may reflect the relative age of the vector population, with large numbers of young mosquitoes entering the population and reducing the proportion of infective mosquitoes.

Although *Pf*EIR has been a common parameter used for capturing the intensity of transmission the accuracy of these estimates have been rarely described. Whilst it is relatively easy to generate 95% confidence intervals around the HBR and *Pf*SR, it is unclear how to combine these for the composite *Pf*EIR. Because of concerns about sample sizes and the shape of the distributions, bootstrapping was compared with conventional methods based on the central limit theorem [34]. Both methods gave similar results. Future studies will report on methods using modelled statistical distributions.

Criteria for selecting a protocol for entomological methods are based on many criteria including costs, ethics, precision, and accuracy. One motivation for using light traps rather than human-landing catches is that light traps are considered to be more ethically acceptable than human-landing catches since they do not require a person to be exposed to biting mosquitoes. In reality those engaged in collecting mosquitoes off their exposed limbs should be employed from the local area and put on strict malaria prophylaxis so that the risk of malaria is much less than normal. A recent study showed that collectors provided with prophylaxis had a 97% lower malaria incidence compared with non-collectors, illustrating that collectors’ risk of malaria can be considerably reduced [35]. Nonetheless, landing catches can be unreliable since collectors may fall asleep during collections, especially if there are few mosquitoes attempting to feed. Collections can also be biased because the number of mosquitoes collected is dependent on the attractiveness of the human to mosquitoes and on the ability of a collector to catch mosquitoes. Human-landing collections are also expensive, require continual supervision, and are difficult to do on a large scale. Although human-landing catches are considered the gold standard, they may not be the best sampling tool because they tend to under-sample mosquito numbers, especially when mosquito numbers are low.

In this study CDC light traps were shown to be a reasonable alternative to human-landing catches in these study sites, with one light trap collecting nearly three times as many mosquitoes as with one human bait. It can be seen from Table 1 that in most places light traps tend to overestimate collections made using human collectors off their exposed limbs. Light traps are a standardized method of sampling where the catch is not dependent on the skill of the operator. Most people like having a small light in the house at night so there are rarely problems with compliance. A known problem is that these traps are poor at collecting mosquitoes outdoors, they are relatively expensive and the batteries need continuous recharging and changing after two years of continual use. Pyrethrum spray catches have been used to estimate transmission of malaria [36] and in this study counts from knock-down collections combined with exit traps were also shown to be useful for measuring biting rates. These are a simple and relatively inexpensive method for sampling mosquitoes, although they are subject to collector bias and moving a large number of exit traps between sites can be difficult.

Recently the Ifakara B tent trap has been developed where a human sleeps protected inside a small canvas tent as “bait” [37,38], again showing a strong correlation with human-landing catches [39]. The tent traps clearly can be an effective sampling tool but they need to be tested in a variety of situations before they are widely endorsed. However, they probably estimate only the indoor vector biting population since they resemble a small house and they are likely to prove unpopular in area where it is hot at night. Moreover, whilst they are relatively cheap to produce and can be manufactured locally, the costs of hiring a sleeper each night does increase the expense of this tool.

Whilst there has been a move to finding alternatives to human-landing catches over the past decades it is important that this method of sampling is retained for specific purposes. Specifically, whilst efficient tools exist for sampling mosquitoes indoors few efficient methods exist for sampling mosquitoes outdoors. This is important since in some parts of Africa the massive rollout of LLINs has led to dramatic reductions in *An. gambiae s.s.* populations that are strongly endophilic, with transmission being maintained at lower levels by residual populations of the more exophilic vector *An. arabiensis* or newer mosquito species [40]. Odour-baited traps are currently being developed which may be useful for collecting outdoor biting vectors [41,42].

## Conclusions

The present study shows that there was a strong correlation between collections made with human-landing catches and either light traps or knock-down collections and exit traps combined. No other studies could be found that compared human-landing catches with knock-down catches and exit traps together. These results indicate that knock-down catches combined with exit traps were consistent with human-landing catches, although the results from Jinja indicate that they may not be so effective where vectors occur in low densities. Thus this method represents an alternative to light traps. Nonetheless, whilst this technique may be cheaper to operate than light traps, exit traps are bulky and therefore difficult to transport. They are also subject to collector bias.

## Abbreviations

PfEIR: *P. falciparum* entomological inoculation rate; HBR: Human biting rate; PfSR: *P. falciparum* Sporozoite rate; LLIN: Long-lasting insecticidal net.

## Competing interests

The authors declare that they have no competing interests.

## Authors’ contributions

MK, DLS, RH, MRK, SGS, GD, and SWL conceived and designed the study. MK, RH and SWL participated in the data collection. DLS, RK, GL, BG, GD, and SWL participated in the management and analysis of the data. All authors participated in the writing of the manuscript. All authors read and approved the final manuscript.
